# The use of amino acid formulas in pediatric patients with allergy to cow’s milk proteins: Recommendations from a group of experts

**DOI:** 10.3389/fped.2023.1110380

**Published:** 2023-03-22

**Authors:** Carmen Ribes-Koninckx, Jorge Amil-Dias, Beatriz Espin, Manuel Molina, Oscar Segarra, Juan J. Diaz-Martin

**Affiliations:** ^1^ Pediatric Gastroenterology, Hepatology and Nutrition La Fe University and Politechnic Hospital & La Fe Research Institute, Valencia, Spain; ^2^ Emeritus, S. João University Hospital Center, Porto, Portugal; ^3^ Pediatric Gastroenterology and Nutrition Unit, Virgen del Rocio University Hospital, Seville, Spain; ^4^ Department of Pediatric Gastroenterology and Nutrition, La Paz University Hospital, Madrid, Spain; ^5^ Pediatric Gastroenterology, Hepatology and Nutrition Unit, Vall d’Hebron Barcelona Hospital Campus, Barcelona, Spain; ^6^ Pediatric Gastroenterology and Nutrition, Hospital Universitario Central de Asturias, Oviedo, Spain

**Keywords:** cow’s milk allergy, amino acid formula, extensively hydrolysed formula, infant formula, hypo-allergenic formulas, elemental formula

## Abstract

One of the most common food allergies in children is cow’s milk allergy (CMA). In breast-fed infants with CMA, the mother is encouraged to avoid dairy products. If this is not possible, or in formula fed infants, use of hypoallergenic replacement formulas such as extensively hydrolyzed formulas (EHF) is recommended. However, in ∼5% of patients EHFs are not tolerated and/or allergy symptoms can persist. When EHFs are ineffective and in severe forms of CMA, amino acid-based formulas (AAF) should be considered. Six pediatric gastroenterologists with extensive experience in food allergy management reviewed scientific publications and international clinical practice guidelines to provide practical recommendations on AAF. The guidelines reviewed had discrepancies and ambiguities around the specific indications for using formulas as a milk substitute. The panel recommends AAFs as the first therapeutic option in anaphylaxis due to CMA, in acute and chronic severe food protein-induced enterocolitis syndrome, in CMA associated with multiple food allergy, and in cases of eosinophilic esophagitis not responding to an extended exclusion diet or not eating solids. The main benefit of AAF is its absence of residual allergenicity, making it a safe treatment option in severe CMA patients who do not tolerate or respond to an EHF.

## 1. Introduction

The prevalence of food allergy is increasing and cow's milk allergy (CMA) is the most common food allergy in children younger than 3 years (1). The reported incidence of CMA during the first year of life ranges from 2% to 7.5% (2, 3), and decreases to <1% in children 6 years or older (4). The prevalence of CMA is around 0.5% in infants who are exclusively breast-fed (5). CMA occurs because β-lactoglobulin from cow's milk is excreted in human milk 4–6 h after maternal consumption of cow’s milk (6). More recent data suggest that sensitization of offspring with food allergens may occur earlier (i.e., during pregnancy). Food proteins have been shown to be transferred through the placenta, contributing to the induction of neonatal tolerance (prevention effect) or to the development of allergic responses to maternally transferred allergens (7).

If a child has CMA and is fed a milk formula, they require a milk substitute that is well tolerated while providing the essential nutritional requirements for this specific age (8, 9). Extensively hydrolyzed formulas (EHF) are generally used as the first treatment option in CMA (2, 3, 10–15) and can be derived from either bovine milk casein [extensively hydrolyzed casein formula (EHCF)] or whey [extensively hydrolyzed whey formula (EHWF)]. However, in 5%–10% of patients, the use of an EHF may be associated with persistence of allergy symptoms and/or lack of normalization of nutritional status (16, 17). In these cases, the EHF has to be replaced by an amino acid-based formula (AAF) (3, 10, 13, 18–23). In fact, some experts prefer AAF over EHFs in children with severe gastrointestinal disorders (24–29), severe atopic dermatitis (AD) (11, 21, 29, 30) or failure to thrive (3, 18, 31).

Soy-based formulas (SF) are frequently not tolerated by children with CMA, especially in non-immunoglobulin E (IgE)-mediated CMA (up to 60% of cases). Because they contain isoflavones, SFs are not recommended for infants younger than 6 months of age (3). An additional consideration for SFs is the fact that they contain phytates, which decrease the absorption of minerals and trace elements (3, 32). However, it should be noted that most current SFs contain lower levels of phytates and provide higher calcium, zinc and iron content when compared with an adapted formula (33).

From a clinical perspective, there is no real consensus among the different clinical guidelines/position papers on the use of AAF, which stems mainly from the lack of long-term, randomized controlled trials with large study populations. Moreover, no robust recommendations are clearly provided regarding duration of treatment and treatment follow-up. Thus, the aim of this document is to give practical recommendations on the use of AAF in CMA-related disorders in pediatric patients, based on the available evidence but also on the experience of a group of pediatric gastroenterology experts.

## 2. Materials and methods

The literature search strategy and the methodology for the literature analysis are described in the supplementary materials (Supplementary Table S1 and Figure S1). An expert panel was formed that consisted of six pediatric gastroenterologists with extensive experience in the management of food allergy and related situations. The results of the literature search were distributed to the panel for discussion. Following this, the evidence was reviewed, and recommendations created and voted on during the panel discussions. The open discussion process was repeated three times until there was 100% agreement among all panel members.

## 3. Results

### 3.1. Amino acid-based formulas

#### 3.1.1. Definition and main features

An AAF, also known as an elemental formula or elemental monomeric formula, is a nutritionally complete formula for infants that is composed of free synthetic amino acids (AA), as well as fats, carbohydrates as glucose polymers, and micronutrients (23). Their main advantage is the lack of proteic residual allergenicity as they are not derived from cow’s milk protein (CMP) or any other native protein (23). Usually, they contain a variable percentage of fat, like medium chain (MCTs) or long chain fatty acids, to avoid deficiencies in essential fatty acids.

Other advantages of AAFs are that they are absorbed with minimal digestion and are associated with decreased intestinal fecal volume.

Some AAFs include prebiotics, synbiotics, or human milk oligosaccharides, although due to the limited number of studies available, the potential benefit of these additional components has not yet been established. However, a 2021 systematic review that included seven publications of four randomized controlled trials in infants with CMA suggested that an AAF containing synbiotics produces clinical benefits with potential economic implications related to fewer infections and hospitalizations compared with a standard AAF (34).

#### 3.1.2. Benefits of amino acid-based formulas

Formula milk immunogenic proteins are composed of linear epitopes (denatured by enzymatic hydrolysis) and conformational epitopes (denatured by heat). Allergy to conformational epitopes tends to persist longer than that to linear epitopes. Despite being processed by a combination of procedures (heat, enzymatic hydrolysis, ultrafiltration, ultrasound, and gamma irradiation) during which a high degree of hydrolysis of proteins is achieved, EHF may still contain peptides capable of inducing an allergic response (bioactive peptides) (35). Other nutrients (e.g., corn proteins) or additives may also cause an allergic reaction. More recently, it has been recognized that milk lipids, especially sphingolipids, could trigger the inflammatory response in CMA, challenging the theory that allergy is a response induced by antigenic proteins only (36).

While it is considered that a formula composed of peptides with molecular weight lower than 1,200 Da is suitable for most children with CMA, the optimal hydrolysis degree has not been exactly established.

The European Union regulations consider for label purposes that a formula has reduced allergenicity if the content of immunoreactive proteins is less than 1% of the total nitrogen content (32). However, there is no evidence that this limit guarantees the absence of clinical allergenicity. Thus, the European Society of Pediatric Allergy and Clinical Immunology (ESPACI) recommends for the treatment of IgE-mediated CMA to use a formula tolerated by 90% of cases. This means that between 5%–10% of patients may react to an EHF and still require an alternative (i.e., an AAF).

Consequently, although EHCF or EHWF are considered the first therapeutic choice in children with CMA, in severe pathologies such as food protein-induced enterocolitis syndrome (FPIES), eosinophilic disorders or anaphylaxis, an AAF with no allergic capacity may be preferred (37). On the other hand, a consensus document for the management of CMA in the Middle East stated that an AAF should be considered for the diagnostic elimination diet and that a double-blind placebo-controlled oral challenge test should be performed for 2–4 weeks using an AAF in formula-fed infants (38).

There is, however, some controversy concerning the use of AAF in the long term, as peptides of EHF might exert a local and systemic immunomodulatory effect by different mechanisms, accelerating the development of tolerance to CMP (39). Still only a few peptides with immunomodulatory activity have been identified so far and additional studies in humans are needed. Interestingly, an *in vitro* study of protein fractions from five different formulae used for CMA treatment [i.e., EHWF, EHCF, SF, AAF, and hydrolyzed rice formula (HRF)] compared the tolerogenic effects using an infant gut simulated digestion model (40). Different regulatory actions on the tolerogenic mechanisms were elicited by the different protein fractions; the EHCF-derived protein fraction elicited a tolerogenic effect, at least in part, through epigenetic modulation of the Forkhead box 3 (*FoxP3*) gene. This may explain the differences in immune tolerance acquisition that are observed in patients with CMA using EHCF (40).

### 3.2. Amino-acid based formulas as the first therapeutic option

#### 3.2.1. Patients with anaphylaxis to cow’s milk protein

The term “anaphylaxis” defines a generalized or systemic hypersensitivity with acute onset and potentially lethal outcome. Isolated cases of anaphylactic reactions in infants with CMA fed with EHF have been documented, although there are no broad series detailing the prevalence of anaphylaxis linked to EHF (41). In AAF, unlike EHF, there is no residual allergenicity linked to CMA and there is extensive experience on their safety in severe cases.

Even though the last update of Diagnosis and Rationale for Action against Cow’s Milk Allergy (DRACMA) guidelines states that HRF may also be suitable as first choice in IgE-mediated CMA, even in its severe forms (11), other societies state that there is not enough evidence for this recommendation and prefer the use of an AAF as the first option in children with CMP-induced anaphylaxis (3, 13, 14, 42–47).

**Statement:** AAF are safe in children with anaphylaxis to CMP.
**The expert group recommends**: To use an AAF as the first therapeutic option in cases of CMP anaphylaxis.

#### 3.2.2. Amino acid-based formulas in children with eosinophilic gastrointestinal diseases

In the last few years, gastrointestinal disorders involving eosinophilic infiltration of the digestive system have become more common. These may affect any part of the gastrointestinal tract, although they have a different pattern in the esophagus.

##### 3.2.2.1 Eosinophilic esophagitis

In many cases of food-related inflammation, dietary intervention may effectively control the disease (48, 49). However, eosinophilic esophagitis (EoE) is a distinct form of food allergy that is not IgE mediated. The current allergy tests for diagnosing EoE have yielded controversial results and do not provide consistent information for diagnosis or monitoring (26, 50).

The first line of treatment may be tailored to the individual patient, with pharmacological options often preferred to food elimination, as the latter frequently needs multiple endoscopies and biopsies to confirm remission of the inflammation. However, a considerable number of patients will respond to elimination diets. Various models have been proposed (single-, two-, four-, and six-food elimination diets) with variable results. Clearly the step-up approach, beginning with single-food elimination – milk being the most common triggering cause - may avoid multiple endoscopies and reduce the time to identify the exact food causing the disease (51).

Historically, AAF emerged as the elective treatment for the first diagnosed patients with EoE (10, 52–54). While this approach has the highest histological response, being effective in more than 90% of cases, it may not be a sustainable long-term treatment option (42). AAF may have a limited role in severe cases or after failure of a six-food elimination diet. Realistically, it may be of considerable interest in cases of small children not taking solid foods, where rapid improvement is required (2, 55–57).

AAF may also be used to assess whether a patient who is not responding to the six-food elimination can achieve remission on AAF (aeroallergens may also trigger EoE) and proceed with stepwise food reintroduction (24, 58).

AAF have been used to induce remission in patients with cow’s milk-induced EoE. Recently, a small study in adults showed that EHF from CMP may be tolerated by patients with EoE (59). However, this hypothesis has not been addressed in subsequent studies or other patient populations, namely in pediatric patients. It seems reasonable to suggest that AAF may also be considered as the option to complement very restrictive diets in patients who do not respond to more liberal regimens, despite a lack of evidence to formally support this.

**Statement**: Within the dietary approaches to treat EoE, AAF provide the highest efficacy to promote remission.
**The expert group recommends**: To use an AAF to control inflammation in patients with EoE who do not respond to extensive food-elimination diets.

AAF may be useful to treat infants with EoE who do not ingest solid foods.

##### 3.2.2.2. Eosinophilic gastroenteropathy and colitis

Beyond the specific esophageal involvement, any segment and various layers of the stomach and gut wall may also be affected. There is scarce information or guidelines on the diagnostic and management strategies of these forms of eosinophilic-mediated inflammation, which may also involve the esophagus in some cases (19, 60, 61).

In up to 40% of cases, there may be spontaneous resolution of the condition (62). Treatment options depend on the known coexistence of atopy and food allergies. In these patients, sequential elimination diets, such as those used in EoE, may be applied. One possible algorithm is to use AAF to induce remission and then proceed with sequential reintroduction of foods to assess tolerance. In the case of failure or non-feasible dietary strategy, then steroids or other immunosuppressor therapy may be needed (63).

A specific type of allergic proctocolitis occurs frequently in infants exposed to cow’s milk-based formula, but also in exclusively breastfed babies through fragments of ingested proteins excreted into the mother's milk. This type of colitis is usually associated with blood in stools or colic. Eosinophilic infiltration can be observed in biopsies (though rarely needed). Transient elimination of CMP (through an elimination diet of the nursing mother or EHF) usually results in rapid resolution of all symptoms. In rare instances, an AAF may be needed to improve the proctocolitis (3).

**Statement:** In patients with eosinophilic gastroenteritis who do not respond to elimination diets, AAF may be used as part of a diagnostic strategy to induce remission before stepwise re-introduction of foods.
**The expert group recommends**: To use an AAF to induce remission in patients with eosinophilic gastroenteritis who do not respond to elimination diets.

The duration of AAF must be adapted to the individual patient according to presenting symptoms, nutritional impact, and results from allergy tests.

To use an AAF to revert allergic proctocolitis in infants who do not respond to an EHF.

#### 3.2.3. Cow’s milk protein-induced enterocolitis syndrome

Cow's milk is the most frequent trigger of FPIES (28). Recently, a multicenter retrospective study in Spain of 44 infants with cow's milk-related FPIES reported the use of an EHF in nearly 70% of cases and an AAF in approximately 21%. However, in severe forms, 33% received AAF while the percentage was 18% in mild/moderate forms (24).

Similar percentages were reported in 104 children with cow's milk FPIES, where 62% tolerated an EHF, while 39% needed an AAF (64).

The recommendations for cow's milk-related FPIES vary depending on the guidelines. Thus, the European Academy of Allergy and Clinical Immunology (EAACI), British Society for Allergy & Clinical Immunology (BSACI), and DRACMA 2016 recommend AAF as the first therapeutic option (11, 13, 46). On the other hand, the European Society for Pediatric Gastroenterology, Hepatology and Nutrition (ESPGHAN), DRACMA 2010, and North American consensus on FPIES 2017 consider that EHF could be the first option, reserving AAF for selected cases, with greater severity and/or failure to thrive (3, 28, 30). The clinical criteria used to identify severe cases of acute or chronic FPIES are clearly described elsewhere (28). FPIES has been described in preterm infants. In a case series of six preterm newborns with FPIES (65), most infants failed to recover with an EHF, while all responded to an AAF. All cases of FPIES described were categorized as severe according to the current consensus definitions (28).

**Statement:** There is no generalized consensus about the type of formula (AAF or EHF) to be used as the first therapeutic option when nutritional treatment of cow's milk-related FPIES cases requires the use of a hypoallergenic formula.
**The expert group recommends:** To use an AAF as the first therapeutic option in cases of severe FPIES, both acute and chronic forms.

#### 3.2.4. Patients with multiple food allergies

The term “multiple food protein intolerance of infancy” (MFPI) was coined in 1995 in a series of 18 infants found to be intolerant/allergic to EHF or SF, and other foods. After 2 months with an AAF, a double-blind challenge with the formula previously used produced the same original clinical symptoms in 12 of the 18 infants: in 2 of them, immediately and, in the rest, over the next 7 days (66). Later, infants allergic to EHF and other foods were treated safely and effectively with an AAF, with symptom resolution in less than 2 weeks (23, 67).

In 1999, DJ Hill described the natural history of 18 infants who received AAF after being intolerant to EHF. These infants were followed-up (including allergic and anthropometric assessment/documentation) until the age of 3 years. Foods with a positive skin prick test were tested annually, while foods with a negative skin prick test were introduced sequentially every 2 or 4 weeks. Non-tolerated foods were tested every 6 months. Most infants tolerated low-allergenic foods (cereals, vegetables, fruits, and meat) by the age of 2 years. Infants who were not yet tolerating the initial formula at the age of 1 year were tested annually. Only 3 of the 18 infants still required an AAF at the age of 3 years. Sensitized infants (elevated specific IgE) had longer mean times to reach tolerance. All infants who had failure to thrive reached normal growth at the age of 2 years with an AAF (68).

Despite evidence based only on a few cases, scientific societies such as ESPGHAN or BSACI recommend an AAF as a first option in multiple food allergy or severe enteropathy (3, 12).

Recently, in 24 infants with MFPI, AAF was found to be safe and effective both at an allergic and nutritional level (27). This raises the question whether we should continue calling this an MFPI entity or classify it within the broad spectrum of non-IgE-mediated CMA, considering the new food allergy guidelines (2).

**Statement:** AAF has proven efficacy and safety both at an allergic and nutritional level in situations of multiple food allergies.
**The group of experts recommends:** To use an AAF as the first therapeutic option in cases of allergy to CMP associated with multiple food allergies.

#### 3.2.5. Patients with moderate/severe nutritional impairment

There has always been a great concern for CMA patients with associated nutritional involvement, and several publications compare the efficacy and safety of both AAF and EHF in this scenario (31, 69, 70).

In a study by Isolauri and colleagues involving 45 infants with 9 months of follow-up, growth rates were higher for AAF, with a significant increase in height (*p* = 0.006) and a positive trend in weight gain (*p* = 0.09) compared with EHF. Based on this, the authors recommended an AAF to preserve growth in infants with multiple food allergies (69).

In 73 infants after a 6-month follow-up, Niggemann and colleagues found growth rates were higher for AAF with a significant increase in height (*p* < 0.04). The authors concluded that AAF could be a beneficial alternative in severe cases of CMA (31).

In another study in 40 infants who were followed-up for 24 months, there was a significant increase (*p* < 0.05) in weight for height in the group treated with EHF, but there was no significant differences between treatment groups (EHF vs. AAF) (70).

A systematic review of 20 studies on the clinical efficacy of AAF (remission of symptoms and outcomes related to failure to thrive), reported similar safety and efficacy in some uncomplicated forms of CMA. However, in complex digestive manifestations or multiple food allergy, AAF was superior to EHF or SF (21).

The guidance from the different scientific societies is not consistent for patients with CMA and concomitant affected nutritional status. Both ESPGHAN and BSACI recommend the use of AAF as a first option in severe enteropathy with hypoproteinemia and failure to thrive. However, DRACMA guidelines recommend continuing an EHF and switching to an AAF if failure to thrive persists (3, 11, 12).

**Statement**: Most of the studies in allergic patients with affected nutritional status report similar weight gain when receiving EHF or AAF.
**The expert group recommends**: To use an AAF in cases of severe nutritional impairment or in the absence of a progressive nutritional recovery under previous EHF treatment.

#### 3.2.6. Patients with atopic dermatitis

AD in children is associated with food allergy in 33%–54% of cases, cow’s milk being one of the food products most frequently involved (13, 69). Conversely, up to 50% of patients with CMA present with AD (55). The use of an EHF or an AAF not only avoids the implicated proteins, but will exert an immunomodulatory effect either direct or through microbiota modifications; the use of EHF has been associated with a decrease in tumor necrosis factor, indicating a reduction in intestinal inflammation (71). Children with severe AD often have nutritional impairment due to several factors: chronic illness *per se*, loss of protein (72), maintained allergic inflammation (73), loss of sleeping hours and impaired release of growth hormone (74), vitamin D deficit, and steroid treatments (31). In these severe cases, both the restriction diet and a delay in clinical improvement can worsen the nutritional status, so there is some debate about the most appropriate treatment (31).

In two randomized multicenter studies including infants (mean age 6 months) with CMA and AD (a total of 45 and 75 patients in each study, respectively), there was a similar 50% decrease in the SCORIng Atopic dermatitis score in all patients on AAF or on EHF after 6 months (31, 75). However, one trial reported that patients had better weight and height gain with AAF (75) and the other study a greater increase only in height with AAF (31); these results were observed despite similar caloric intake in the two treatment groups. The authors concluded that the use of an AAF may be a good alternative to treat severe CMA (31).

In 16 children with CMA who did not improve on an EHF, after switching to an AAF there was remittance of non-cutaneous symptoms in all patients, while eczema improved in 4 out of 5 patients (67). Similarly, after EHF was replaced by an AAF in 10 children with severe persistent AD, clinical remission was obtained (76).

A systematic review reported several isolated cases not responding to an EHF that resolved once switched to an AAF, one of them with AD (21).

Due to the lack of evidence, the guidelines make recommendations based on clinical practice. DRACMA (2010 and 2016) recommends an EHF as the first choice in AD and an AAF as the second option (11, 30). The British guide for CMA management in primary care recommends the use of AAF in severe AD, moreover if there is nutritional involvement, AAF could be used as an alternative to exclusive breastfeeding or formula feeding (77). The evidence and recommendations for exclusively breastfed children with eczema are discussed in Section 3.4.

**Statement:** AAFs are mostly considered an option in severe AD cases and/or nutritional involvement or as a rescue formula when there is a lack of response to EHF.
**The expert group recommends:** To use an AAF for infants with AD who are formula-fed and do not respond to EHF.

To use an AAF in formula-fed infants who present severe AD with nutritional impairment (until nutritional recovery).

#### 3.2.7. Patients with intestinal failure

Intestinal failure is the result of a number of different diseases causing chronic dependency on parenteral nutrition to achieve a proper electrolyte and nutrient balance, as well as optimal growth. The North American Society for Pediatric Gastroenterology, Hepatology & Nutrition (NASPGHAN) defined intestinal failure as the need for parenteral nutrition for more than 60 days due to illness, dysfunction, or intestinal resection (78).

The most common cause of intestinal failure in children is short bowel syndrome, in which there is a decrease in the absorptive capacity of the intestine due, in most cases, to a surgical resection.

There are few data in the literature to safely recommend the most appropriate oral formula for these patients, but general agreement is that feeding should begin as soon as possible, even at trophic volumes.

The risk of developing CMA is estimated to be 2- to 4-fold higher in these patients, especially among infants with surgical short bowel syndrome (79).

Therefore, along with the absorptive limitation, it seems advisable not to use whole protein formulas, especially in younger infants. However, the choice between an EHF or an AAF is more controversial. On the one hand an AAF is more readily absorbed, and thus could be more beneficial in patients with significant functional limitation of the remaining intestine, or in younger patients and in those with a shorter remaining intestine. However, there are no studies that demonstrate this, except for some anecdotal published cases that improved after switching from EHF to AAF (80).

On the other hand, it has been shown that the presence of nutrients in the intestinal lumen is a key factor promoting intestinal adaption and digestive autonomy, favoring the use of EHF, as it is more complex than AAF. Thus, some authors would recommend starting breastfeeding or even a formula with whole CMP, switching to an EHF if the previous one is not tolerated, and reserving the use of AAF if the EHF fails (81, 82).

Finally, it should be noted that there are no high-quality data available to recommend the use of EHF or AAF in intestinal failure due to intractable diarrhea or other serious functional digestive disorders.

**Statement**: In infants with short bowel syndrome who are not breastfed, using an EHF promotes intestinal adaptation and may help patients achieve intestinal autonomy.
**The expert group recommends**: To use AAF formula for patients who are EHF intolerant and in the initial phases of treatment in patients with less digestive and /or absorptive capacity.

### 3.3. Amino acid formulas as a therapeutic option to replace an extensively hydrolyzed formula

Children with CMA may not achieve partial or total control of their symptoms with an EHF. They may require an AAF due to adverse reactions not mediated by immune responses (most frequently, sugar intolerances), possible immune reactions against other formula components (especially lipids or glycoproteins), and residual allergenicity of CMP peptides present in the EHF (19, 20, 23, 28, 66, 67, 83, 84).

#### 3.3.1. Residual allergenicity of extensively hydrolyzed formulas

The term “hypoallergenic formula” was used for the first time in 1991 to refer to those formulas tolerated by more than 90% of people with a confirmed CMA (85). Subsequently, van Beresteijn (86) and Plebani (87) demonstrated through enzyme-linked immunosorbent assay (ELISA) the concept of residual antigenicity of EHF in those cases of CMA in which the formula was not tolerated. van Beresteijn detected CMP-specific IgE and IgG antibodies in the serum of allergic patients against different intact protein milk formulas. They also detected antibodies against hydrolyzed protein formulas, although to a lesser extent (86).

Thus, the tolerance pattern of a hypoallergenic formula is thought to be determined by its residual allergenicity. Immunogenic material related to casein can be found in EHF, suggesting that either casein removal from whey is insufficient or the hydrolysis process shows hidden antigenic determinants of casein. The presence of other antigenic proteins, such as β-lactoglobulin, cannot be excluded.

**Statement:** EHF can contain residual CMP epitopes capable of inducing an allergic response.

#### 3.3.2. Treatment failure with an extensively hydrolyzed formula

Failure of treatment with an EHF is a broad term and is used to describe the persistence of all or some CMA symptoms in patients who receive this formula. While all societies and consensus recommend AAF as the alternative for those cases, establishing treatment failure may be difficult. More recently, HRF has emerged as an option, both in cases of EHF treatment failure as well as first-line treatment of CMA; however, the lack of HRF availability worldwide plus limited supporting evidence has prevented a generally accepted consensus on its use (88–90).

In IgE-mediated CMA, it is easy to determine symptom resolution. However, in non-IgE-mediated CMA, symptom improvement is progressive and resolution needs more time. In these cases, recommendations on the timeframe in which symptoms should resolve are very broad (2–6 weeks) and vary according to the type of clinical entity (3, 6, 11, 28, 42, 44, 45, 55). Thus, in cases with acute symptoms and no nutritional involvement (as in acute FPIES) it is between 24 and 72 h, while in cases with minor gastrointestinal symptoms (e.g., gastroesophageal reflux), as well as in those with progressive and gradual onset (e.g., enteropathy or AD due to CMA) and nutritional involvement, full resolution can take between 2 and 4 weeks (14, 53, 91). Complete control of symptoms beyond these intervals is considered unlikely. Lozinsky and colleagues reported the results of a study in 131 children with non-IgE-mediated food allergy in which 98% of patients improved 4 weeks after starting the diet, while only 2% did so after 8 weeks (92).

In the majority of cases with symptoms persisting beyond the theoretical period established for resolution, switching to an AAF leads to complete control of symptoms and to an improvement in the nutritional status (1, 20, 22, 23, 27, 66, 67, 83, 84). However, only in some studies was the existence of a reaction to EHF confirmed by a subsequent challenge test (23, 66, 67, 83, 84).

In 28 children with non-IgE-mediated CMA with persistence of gastrointestinal symptoms and/or low weight gain on an EHF (mean treatment time 40 days; range 10–173 days), switching to an AAF achieved improvement in 25 patients after 2 weeks. After the reintroduction of EHF, symptoms reappeared in 17 children but not in 8 (32%). These cases may reflect an insufficient therapeutic response to EHF with persistence of some intestinal inflammation that prevented optimal nutrient absorption, or simply that initial maintenance of EHF was not long enough (23). After symptom improvement was achieved by AAF, EHF was tolerated.

**Statement:** Treatment failure with EHF is considered if part or all the initial symptoms persist after more than 2 weeks or there is lack of nutritional recovery, even if the dominant symptom may have resolved. Symptom control with an EHF in children with non-IgE-mediated CMA can take 2–4 weeks but is rare after this time period. AAF achieves control of symptoms in most children where there is a failure with an EHF.
**The expert group recommends**: To assess the response to an EHF 2–4 weeks after starting treatment.

To employ an AAF when there is a treatment failure with an EHF.

To use an AAF when nutritional impairment persists with an EHF, regardless of an improvement of other symptoms.

### 3.4. Infants who develop symptoms with exclusive breastfeeding

Small amounts of CMP are detected in breast milk of lactating women, hours or even days after ingestion (93). However, only in some children do these CMPs trigger the immune mechanism of allergy and, in these cases, mothers should be encouraged to continue breastfeeding while avoiding CMP in their own diet. There is a hypothesis that infants who develop symptoms while exclusively breastfeeding could more likely react to peptides present in EHF, but this has not been confirmed (94).

Two societies make specific recommendations for breastfed infants with severe symptoms. BSACI suggests using an AAF to achieve a more rapid stabilization of the child’s condition for those patients with severe eczema who do not respond after CMP has been eliminated from their mother’s diet (46); ESPGHAN recommends the use of an AAF for 2 weeks in breastfed infants with severe symptoms [e.g. severe AD or allergic (entero) colitis complicated by growth faltering and/or hypoproteinemia and/or severe anemia] (3). These recommendations are not evidence based since, for ethical reasons, there are no studies comparing breast milk vs. AAF, and data on severe cases in exclusive breastfed infants are scarce and conflicting (Table 1) (95–102). More recently, the Global Allergy and Asthma European Network (GA^2^LEN) recommended for infants with CMA who need a breastmilk alternative to use either an EHF or AAF (moderate certainty), although they state that the cost of AAF may prevent it being the first treatment choice (89). Some children do not respond to the mother’s exclusion diet, either due to the difficulty in maintaining a strict CMP-free diet or to reactivity against other allergens (the most frequent being soy, egg, cereals, and nuts). Maternal avoidance of one or more of these foods may adversely impact the nutritional status of the lactating mother and should always be supervised by a dietitian. Moreover, avoidance of multiple foods should be limited to short period of time because a restricted maternal diet may compromise the maintenance of breastfeeding due to the difficulties for the mother in complying with these restrictions (103).

**Table 1:** Severe food protein-induced enterocolitis syndrome (FPIES) in infants exclusively breastfed.

Author, Year (ref)	Clinical Features	Triggers of acute reactions In OCT	Treatment
Monti, 2011 (98)	1 month-old infant, Chronic FPIES	Cow’s milk-based formula CMP through breastfeeding after maternal ingestion	EHF 1 week Reintroduction of breastfeeding (milk and dairy products were avoided but baked products that might contain heated milk were permitted) supplementation with EHF From 2 months of age onwards: Breastfeeding (CMP free diet) and supplementation with EHF
Nomura, 2011 (99)	Three infants, Bloody diarrhea, vomiting	Cow’s milk-based formula CMP through breastfeeding after maternal ingestion Rice and soy	Cessation of breastfeeding Formula type not specified
Tan, 2012 (101)	5 month-old infant, Acute FPIES	Soy formula Soy through breastfeeding after maternal ingestion	Breastfeeding (soy free diet but processed foods that might contain traces of soy protein were permitted)
Miceli Sopo, 2014 (97)	1 month-old infant, Chronic FPIES. AD	CMP breastfeeding after maternal ingestion	Breastfeeding (CMP- and egg-free diet)
	2 month-old infant, Chronic FPIES. AD	Cow’s milk-based formula CMP through breastfeeding after maternal ingestion	Breastfeeding (CMP-free diet)
Kaya, 2016 (96)	15 day-old infant, Chronic FPIES	CMP through breastfeeding after maternal ingestion Cow’s milk-based formula	Breastfeeding (CMP-free diet) and supplementation with AAF
	2.5 month-old infant, Chronic FPIES	Cow’s milk-based formula	Breastfeeding (CMP-free diet) and supplementation with AAF
Vergara Perez, 2018 (102)	2 month-old infant, Acute FPIES	No OCT was performed	AAF 2 weeks Reintroduction of breastfeeding (CMP-free diet)
Ntoumpara, 2019 (100)	4 month-old infant, Chronic FPIES	Partially HF CMP and soy through breastfeeding after maternal ingestion	AAF
Baldo, 2020 (95)	2 month-old infant, vomiting, diarrhea, dehydration, failure to thrive. Raised level of methemoglobin.	No OCT was performed	AAF
	3 month-old infant, vomiting, diarrhea, dehydration, failure to thrive. Raised level of methemoglobin. Eosinophilic infiltration of the duodenal mucosa	No OCT was performed	Breastfeeding (CMP-free diet) and supplementation with AAF

AAF, amino acid formula; AD, atopic dermatitis; CMP, cow’s milk proteins; EHF, extensively hydrolyzed formula; FPIES, food protein-induced enterocolitis syndrome; HF, hydrolyzed formula; OCT, oral challenge test.

However, even after excluding other allergens from the mothers diet, complete resolution of the symptoms is not attained for some patients, and thus, in these cases, AAF could be an option. Although some observational studies report patients were able to achieve control of symptoms after switching to AAF, to date there are no clinical trials comparing the efficacy of AAF vs. EHF. Isolauri and colleagues studied 100 infants with AD during exclusive breastfeeding (73). All lactating mothers modified their diets: eighty of them eliminated several basic foods including cow’s milk, egg, fish, and cereals and 20 eliminated single foods such as citrus fruits. A diagnosis of CMP allergy based on double-blind placebo-controlled challenge was made in 59 patients. The main reason for cessation of breastfeeding was persistence of allergic symptoms, pruritus, and sleep loss in 97% of cases. The intensity and extent of AD and nutritional status improved significantly after weaning from breastfeeding to an AAF. Those with the worst nutritional status were those with more severe AD and, the greater the period was between the onset of symptoms and cessation of breastfeeding, the more significant the risk of poor growth. These data indicate that the persistence of allergy symptoms during exclusive breastfeeding could contribute to an inflammatory process that may interfere with normal infant growth. Latcham and colleagues studied 121 children with multiple food allergies (104). Thirty-six (29.7%) showed reaction with EHF and required the use of AAF. Remarkably, all of them developed symptoms while being breastfed only. In another study, Lake and colleagues described a cohort of 95 infants who developed allergic proctocolitis during exclusive breastfeeding (105). Eleven did not improve on a maternal elimination diet and stopped breastfeeding. EHF led to full improvement in 7 patients, while 4 patients (all of them with eczema) required an AAF.

Sometimes exclusively breastfed infants with proctocolitis do not have a complete resolution of symptoms despite maternal dietary restrictions; intermittent presence of small amounts of mucus or rectal bleeding may persist but without clinical implications. Most tend to resolve in a variable period of time, and usually with no need for greater restriction of the mother’s diet or weaning from breastfeeding to a formula (105–107).

Two reviews focusing on the appropriate use of AAF indicate that the choice of an AAF instead of an EHF should be based on a combination of symptoms, rather than a single condition or specific symptom. Children more likely to benefit from an early use of AAF mostly show complex diseases, with a combination of overlapping symptoms (including growth faltering) and multiple food allergies, and often had these symptoms already while breastfeeding (21, 55).

**Statement:** AAF can achieve control of symptoms in those children exclusively breastfed who do not respond (partially or totally) to a maternal exclusion diet.
**The expert group recommends:** To use an AAF in exclusively breastfed infants with severe symptoms (digestive and/or skin), especially when accompanied by failure to thrive.

To use an AAF in infants who do not respond to a maternal exclusion diet, especially if they are nutritionally impaired.

### 3.5. Follow-up of a patient on amino acid formula – when to replace it?

CMA tends to be self-limited and tolerance regained after temporary elimination. However, there is currently insufficient evidence to recommend the ideal time to re-assess for protein tolerance. An expert consensus on CMA recommends maintaining the formula that has been shown to be effective in reversing the symptoms for 12 months, or at a minimum for 6 months (14). In severe forms of allergy, it is appropriate to test for specific IgE: if negative, challenge with CMP may be considered after at least 6–12 months of elimination diet. In cases of negative serum IgE, 6–12 monthly reassessments are desirable (13). In any case, the timing of reassessment depends on the patient’s age at diagnosis; for example, if the diagnosis is made in the first 6 months of life, as in the majority of cases, it is likely that reassessment will not be required for a further 6 months (i.e., not before 1 year of age) (3, 66).

The duration of the AAF should be limited if this is the single source of nutrition. Although some studies have shown AAF to be safe and associated with appropriate growth, it should be taken into consideration that the child may need to start solid food and establish diversity in their diet (18, 108).

The challenge with CMP may be made with a standard formula in infants or even cow’s milk in older children, as there is insufficient evidence to support the use of EHF as more beneficial. In children older than 3 years, it is recommended to use lactose-free milk to avoid a possible effect of lactose in a child with lactose malabsorption. In case of a severe previous reaction, then a careful stepwise ingestion must be performed in a hospital environment (3).

**Statement:** Children who need AAF due to allergy may be tested for tolerance after a period of CMP elimination. Duration of the diet and ideal moment for food challenge depends on the initial clinical presentation and allergy tests.
**The expert group recommends:** In patients receiving an AAF, duration of the elimination diet must be tailored individually, according to presenting symptoms, nutritional condition, and allergy tests.

Challenge with CMP may be done with a formulated standard, or whole milk (if under medical supervision), using an exposure specific protocol.

### 3.6. Controversies in the use of amino acid formulas

#### 3.6.1. Possible limitations

Advantages and limitations of the AAF vs. EHF are shown in Table 2. AAFs are expensive and have an unpleasant taste (11). A study in which a group of adults evaluated different organoleptic and sensory characteristics of different therapeutic formulas, showed that AAFs had poorer palatability and a worse odor than EHWF (109).

**Table 2:** Advantages and limitations of amino acid and extensively hydrolyzed formulas.

	EHF	AAF
Similarities	High cost Unpleasant taste Elevated osmolarity Nutritionally adequate
Differences	May cause allergic response in 5%–10% of children with CMA	In 100% of children with CMA, they do not trigger an allergic mechanism related to CMP
	Presence of peptides with residual antigenic capacity (immunomodulatory effect)	Absence of bioactive peptides (does not produce immune modulation)

AAF, amino acid formula; CMA, cow’s milk allergy; CMP, cow’s milk proteins; EHF, extensively hydrolyzed formula.

On the other hand, AAFs generate a higher renal solute load and have a lesser effect on epithelial maturation and intestinal enzymatic activity (11). The expected evolution of CMA is that tolerance develops over time. It has been speculated that certain strategies based on stimulation of the immune system by probiotics and bioactive peptides derived from casein are capable of accelerating tolerance acquisition. The absence of allergenicity of the AAF makes them safer in treating allergy, but due to the lack of immunogenic peptides, there is no stimulating effect on the immune system. In a recent non-randomized study that included 260 children with CMA (110), those receiving EHCF with or without *Lactobacillus rhamnosus GG* developed tolerance more frequently after 12 months of treatment than those receiving an AAF (79%, 44% and 18%, respectively). In a similar prospective cohort study of 365 infants with IgE-mediated CMA, a lower incidence of atopic manifestations and a greater rate of immune tolerance acquisition at 36 months was observed in children treated with EHCF containing the probiotic *L. rhamnosus GG* compared with those receiving EHWF, RHF, SF, or AAF (111).

AAFs are more expensive than EHFs. Although there are no specific data on the economic impact of the use of such formulas, a retrospective, non-randomized, US study concluded that the use of an EHF with a probiotic is less costly compared with an EHF without a probiotic or an AAF (112). However, the authors acknowledge important limitations of the study. Comparable results were observed in a similar study in Spain (113). A cost-effectiveness analysis conducted in infants with CMA in the United Kingdom (and not easily applicable to other countries) concluded that AAF was less cost effective than EHF (with or without probiotics) and led to a lower percentage of patients developing tolerance at 3 years after diagnosis (114).

#### 3.6.2. Nutritional deficits related to the use of amino acid formulas

Studies generally show that AAFs are safe, and infants who receive AAF achieve adequate growth and weight gain (10, 14, 15, 20, 23, 67, 115–117).

In most published studies, AAFs were effective in ensuring growth in children with CMA and other gastrointestinal disorders (118), even though children with CMA have greater risk of failure to thrive (119). Observational and randomized multicenter studies including about 700 patients reported normal growth in children with food allergy treated with an AAF (118).

Children with CMA are at risk of consuming less than the recommended amount of calcium (120). However, in a recent study, 66 infants with CMA receiving AAF for 16 weeks had a normal status for different minerals and the vast majority had mineral intake within the recommendations (121).

In a non-systematic review of prospective randomized studies on growth in healthy infants (from <15 days to at least 4 months of age) fed with an EHF (5 studies) or an AAF (4 studies), no accelerated growth was observed in infants fed with an AAF even though these had a higher protein content (122).

Bone health was evaluated in a retrospective, single-center study in 102 infants and young children on enteral nutrition, 78 on AAF and 22 on EHF. Participants presented multiple comorbidities (e.g., prematurity, cerebral palsy, encephalopathy). Including the 26 infants with complete data, only four met criteria for metabolic bone disease. The authors concluded that it is necessary to control bone metabolism in those children where an AAF is used to cover almost all caloric needs in long term enteral nutrition (123).

**Statement:** AAF are efficient in assuring growth in children with CMA.
**The expert group recommends:** To regularly monitor bone metabolism in children who receive an AAF as their main source of food.

## 4. Conclusions

The main benefit of AAF is its absence of residual allergenicity, so it is a safe treatment option for patients with severe CMA who do not tolerate an EHF. Compared with EHF, AAF are expensive, have a less-pleasant taste, and the high renal solute load of AAF may also limit their acceptance.

The panel recommends the use of an AAF as a first therapeutic option in anaphylaxis due to CMP, in both acute and chronic severe FPIES, in CMA associated with multiple food allergy, and in cases of EoE not responding to an extended exclusion diet or not eating solids. Equally, its use is recommended in infants with AD not responding to an EHF, and in those exclusively breastfed with severe digestive or skin symptoms that fail to respond to a maternal exclusion diet. An AAF is recommended as rescue therapy in severe nutritional impairment or in the absence of nutritional recovery with EHF. Partial or complete persistence of initial symptoms or lack of nutritional recovery after more than 2–4 weeks of treatment with EHF is considered failure of treatment and switching to an AAF is recommended. The duration of treatment with an AAF should be adjusted individually depending on the severity of the initial symptoms and the nutritional impairment of the patient. Overall, in our opinion, AAF are indicated in patients with severe CMA, especially in those with associated nutritional impairment or when EHF treatment failure is established. Due to the limitations of its use, 6–12 monthly reassessment for CMA should be performed, with the timing of reassessment tailored to the individual patient, considering the age at diagnosis, clinical picture, serum IgE results, and nutritional status.
