# Supplementary material for: The use of amino acid formulas in pediatric patients with allergy to cow’s milk proteins: Recommendations from a group of experts
Source: Front Pediatr. 2023 Mar 22;11:1110380. doi: 10.3389/fped.2023.1110380 (PMC10073469; doi:10.3389/fped.2023.1110380)
Supplement: Supplementary file 1 [file Table1.docx]

Supplementary Material

# Supplementary Data

## Search Methods

In February 2019, an electronic search in PubMed was conducted to identify articles focusing on CMA in children, plus the usage of basic formulas and EHF, published from January 1990 to February 2019. A restriction was made by language (English and Spanish), but not by study design. Based on the MeSH terms and related concepts, an advanced text search was conducted with free text, to identify relevant articles by the title or in the summary of the publication.

This search was stratified into four levels:

1. Find articles about non-IgE mediated CMA.

2. Search for articles dealing with the use of elemental formulas or AAF.

3. Search for papers in pediatric patients.

4. Search for papers that discuss the use of EHF and AAF.

The four search chains were combined as shown in Supplementary Table 1, obtaining a total of 158 different items. Additionally, a complementary search in Registro de Medicina en Español (MEDES), using the same search criteria obtained 31 additional publications. Finally, after an extended search in PubMed until 18 January 2023 (using the same criteria as for the first search) a total of 248 papers were considered (see Supplementary Figure 1).

### Analysis of the search results

Two authors (BE and CR) made an initial selection based on the summaries, excluding those with experimental content without clinical application and those related to CMA prevention and clinical cases (Supplementary Figure 1).

The selected 95 items were classified into five clinical domains: 1. AAF as the first therapeutic option in children with artificial feeding, 2. AAF as a second treatment option in children with artificial feeding, 3. indications in infants exclusively breastfed, 4. follow-up of patients undergoing treatment with AAF, and 5. controversies in the use of AAF. The results of the search were subsequently distributed among the authors. In addition to the search results, the authors added 28 articles and clinical guidelines (not included in the initial systematic search) related to the specific topic they were responsible for.

# Supplementary Figures and Tables

## Supplementary Figures


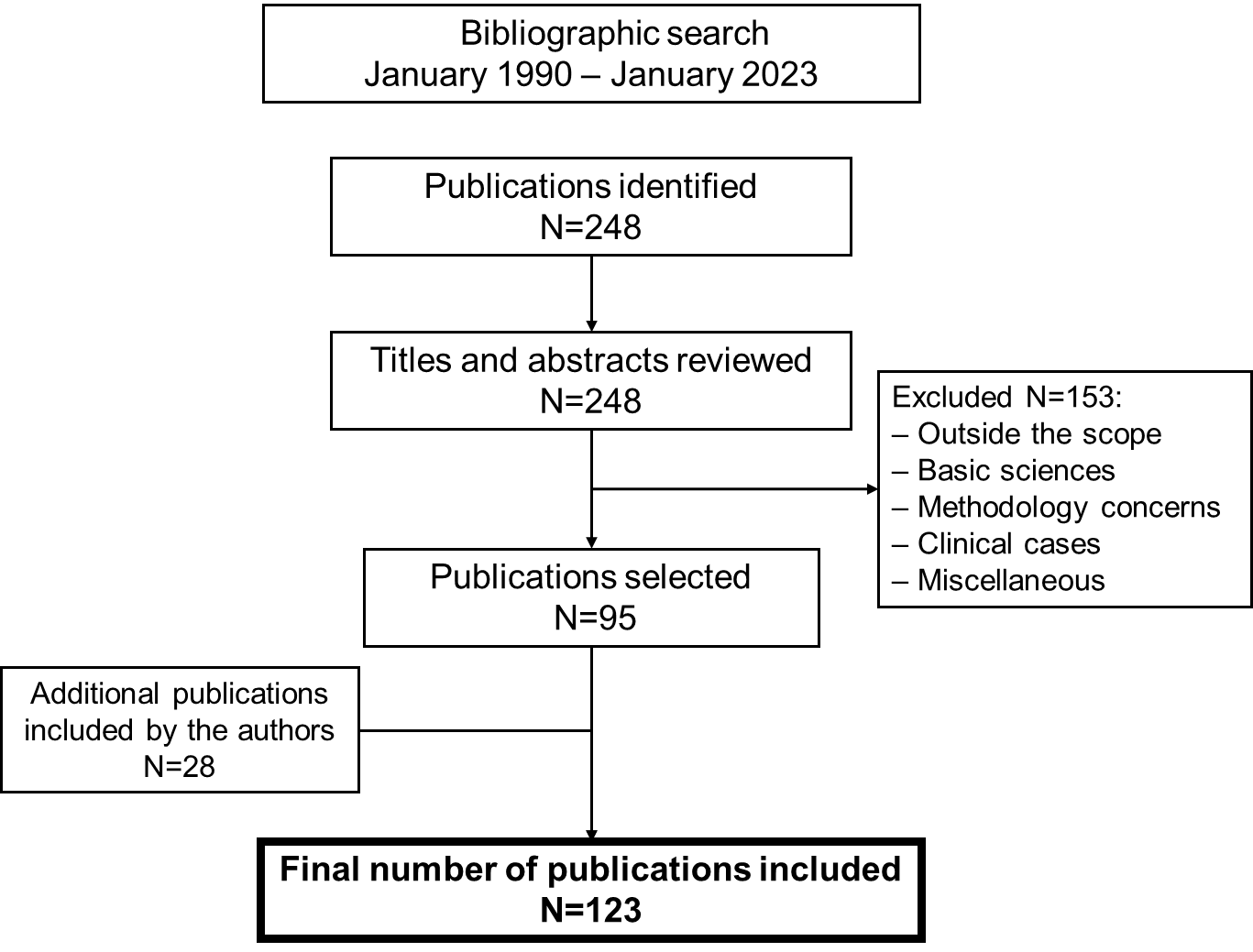


**Supplementary Figure 1.** Summary of search results and final inclusion/exclusions.

## Supplementary Tables

**Supplementary Table 1.** Literature search terms

| 1 | ((((((((((Cow's Milk Protein Allerg*[Title/Abstract]) OR Cow* Milk Protein Allerg*[Title/Abstract]) OR Cow* Milk Allerg*[Title/Abstract]) OR CMPA[Title/Abstract])) NOT ((((((IgE-Mediated[Title]) OR Hypersensitivity Immediate[Title]) OR Immediate Hypersensitivity[Title]) OR IgE Mediated[Title]) OR Mediated IgE[Title]) OR "Hypersensitivity, Immediate"[Majr])))))) AND ( ( "1990/01/01"[PDat] : "3000/12/31"[PDat] ) AND ( English[lang] OR Spanish[lang] ) ))) |
| --- | --- |
| 2 | ((((((((elemental formula[Title/Abstract]) OR elemental feed[Title/Abstract]) OR elemental formulas[Title/Abstract])) OR ((((((((AAF[Title/Abstract]) AND ((formula[Title/Abstract]) OR feed[Title/Abstract]))) OR ((hypoallergenic[Title/Abstract]) AND ((formula[Title/Abstract]) OR feed[Title/Abstract]))) OR ((aminoacid[Title/Abstract]) AND ((formula[Title/Abstract]) OR feed[Title/Abstract]))) OR ((Amino Acid[Title/Abstract]) AND ((formula[Title/Abstract]) OR feed[Title/Abstract]))) OR ((Amino Acid-Based[Title/Abstract]) AND ((formula[Title/Abstract]) OR feed[Title/Abstract]))) OR ((((((((("Amino Acids"[Mesh]) AND "infant formula"[Mesh]))))) OR (((("Amino Acids"[Mesh]) AND "Milk Hypersensitivity"[Mesh])))))))))))) |
| 3 | (((((((((child*[Title/Abstract]) OR newborn*[Title/Abstract]) OR infant*[Title/Abstract]) OR adolescent*[Title/Abstract]))))))) AND ( ( "1990/01/01"[PDat] : "3000/12/31"[PDat] ) AND ( English[lang] OR Spanish[lang] ) )))) |
| 4 | ((((((((((((semi-elemental[Title/Abstract]) AND ((formula[Title/Abstract]) OR feed[Title/Abstract])))) OR Extensively hydrolyzed[Title/Abstract]) OR Extensively hydrolysed[Title/Abstract]) OR EHF[Title/Abstract])) OR protein hydrolysat*[Title/Abstract])) AND ((((((((formula[Title/Abstract]) OR amino acid*[Title/Abstract]) OR aminoacid*[Title/Abstract]) |
| Combined | #1 OR #2 AND #3 AND 4 |
